# Stability of Delay Hopfield Neural Networks with Generalized Riemann–Liouville Type Fractional Derivative

**DOI:** 10.3390/e25081146

**Published:** 2023-07-31

**Authors:** Ravi P. Agarwal, Snezhana Hristova

**Affiliations:** 1 Department of Mathematics, Texas A & M University-Kingsville, Kingsville, TX 78363, USA; 2 Faculty of Mathematics and Infromatics, Plovdiv University, Tzar Asen 24, 4000 Plovdiv, Bulgaria; snehri@uni-plovdiv.bg

**Keywords:** Hopfield neural networks, delays, Riemann–Liouville type fractional derivative, Lyapunov functions, Razumikhin method

## Abstract

The general delay Hopfield neural network is studied. We consider the case of time-varying delay, continuously distributed delays, time-varying coefficients, and a special type of a Riemann–Liouville fractional derivative (GRLFD) with an exponential kernel. The kernels of the fractional integral and the fractional derivative in this paper are Sonine kernels and satisfy the first and the second fundamental theorems in calculus. The presence of delays and GRLFD in the model require a special type of initial condition. The applied GRLFD also requires a special definition of the equilibrium of the model. A constant equilibrium of the model is defined. An inequality for Lyapunov type of convex functions with the applied GRLFD is proved. It is combined with the Razumikhin method to study stability properties of the equilibrium of the model. As a partial case we apply quadratic Lyapunov functions. We prove some comparison results for Lyapunov function connected deeply with the applied GRLFD and use them to obtain exponential bounds of the solutions. These bounds are satisfied for intervals excluding the initial time. Also, the convergence of any solution of the model to the equilibrium at infinity is proved. An example illustrating the importance of our theoretical results is also included.

## 1. Introduction

Stability properties of Hopfield neural networks modeled by various types of derivatives have been studied in the literature. The applied different types of derivatives and their properties could adequately describe different behavior of the dynamics of the neurons. For example, the fractional derivatives have typical memory properties, the Riemann–Liouville type of fractional derivatives have a singularity at the initial time point, and they can model some abnormalities in the behavior of the neurons. We can refer to [1] for Caputo fractional derivative, [2] for conformable fractional derivative, ref. [3] for ordinary derivatives and time-varying delays, and refs. [4,5] for Caputo fractional derivative and delays. In the case when Riemann–Liouville fractional derivative is applied also, several results about the study of stability are published (see, for example, refs. [6,7,8,9] for delays, ref. [10] for random impulses). A good review of the neural networks with applied classical fractional derivatives is given in [11].

In the last few decades, many different definitions of fractional integrals and derivatives have been proposed. One of the ways to generalize the classical ones is to use Sonine kernels (see, for example [12]). The general fractional integrals and derivatives of Riemann–Liouville type were introduced and studied by Luchko [13]. Applying Sonine kernel $h_{\alpha }\left(t\right)=\frac{t^{\alpha -1}}{\Gamma (\alpha )}$, the classical Riemann–Liouville integral (RLI) and Riemann–Liouville fractional derivative ( RLFD) could be written in the following form:

$$I^{RL}f\left(t\right)=\int_{0}^{t}h_{\alpha }\left(t-s\right)f\left(s\right)ds,\;t>0,$$

$$D^{RL}f\left(t\right)=\frac{d}{dt}\int_{0}^{t}h_{1-\alpha }\left(t-s\right)f\left(s\right)ds.$$

Note that the kernel $h_{\alpha }\left(t\right)$ satisfies the classical Sonine condition

$$\left(h_{\alpha }*h_{1-\alpha }\right)\left(t\right)=\left\{1\right\},\;t>0,\;\alpha \in \left(0,1\right).$$

Recently, in [14,15], an integral and two types of derivatives of Caputo and of Riemann–Liouville type, respectively, were introduced and they were called generalized proportional fractional integral and derivatives. The main characteristic of this derivatives is the exponential kernel in the corresponding integrals. The main disadvantage of these type of integrals and derivatives is the absence of mutually inversebility. For these derivatives, the first and the second fundamental theorem is not satisfied. In this paper, based on the ideas of the fractional integrals and derivatives with Sonine kernels, given and studied in [12], we will define a couple of exponential Sonine kernels. These kernels are a partial case of the studied ones in [16] (see Formulas (7.5) and (7.6)). We will use them to define generalized fractional integral and derivative of Riemann–Liouville type. The main advantage of these types of integrals and derivatives is that they satisfy the first and the second fundamental theorems (see Theorems 4 and 5 [17]); therefore, they form a general fractional calculus. One of their advantages is that as partial cases the classical Caputo and Riemann–Liouville fractional derivatives are obtained. We will prove an inequality for the defined general fractional derivative of complex Lyapunov functions. This inequality will be applied to study stability properties of the delays differential equations. An appropriate initial value problem for a model of neural networks with time-variable delays and distributed delays has dynamics which are modeled by the defined general fractional derivative of Riemann–Liouville type.

We consider Hopfield neural networks with both time-variable delays and distributed delays, and variable in time coefficients and external inputs. The dynamics of the units are modeled by GRLFD. Two types of initial conditions are set up. These initial conditions are connected with the singularity of the applied derivatives at the initial time point which coincides with the lower limit of the integral. The equilibrium of the model is defined in an appropriate way and its stability properties are studied. This equilibrium is deeply connected with the applied derivative.

By employing Razumikhin method and appropriate Lyapunov functions, we obtain several upper exponential bounds of the solutions. The obtained results are valid on intervals excluding the initial time which is a singular point for the solutions. We prove the convergence of the solutions to the equilibrium at infinity. The applied derivative gives us as a partial case the classical Riemann–Liouville fractional derivative, so all obtained results in this paper are a generalization of the Hopfield model with the classical fractional derivative.

The innovations of this article can be described as follows:

- We define generalized fractional integral and generalized Riemann–Liouville fractional derivative based on exponential Sonine kernels which satisfy the first and the second fundamental theorems;
- We prove an inequality for general convex Lyapunov functions with generalized Riemann–Liouville fractional derivative;
- We propose a generalized Hopfield neural network model with both time-variable delays and distributed delays. The dynamic of the units are described by the special generalization of the Riemann–Liouville fractional derivative;
- We define the equilibrium of the studied model. It depends significantly on the delays and the applied type of the derivative;
- We use modified Razumikhin conditions deeply connected with the presence of the exponential kernel in the applied derivative;
- We obtain an exponential bound of the equilibrium of the model. This bound is valid only for intervals excluding the initial time point;
- We prove sufficient conditions for approaching any solution of the model to the equilibrium.

The structuring of the rest of the paper pursues the following scheme. The basic definitions for general fractional integral and derivatives are given in Section 2. The basic inequality for convex Lyapunov type functions with the generalized fractional derivative of Riemann–Liouville type is proved in Section 3. Some stability properties of the solutions of delay differential equations with those defined in the paper fractional derivative are proved in Section 4. These results are based on the application of the modified Razumikhin method. Section 5 is devoted to the study of the generalized Riemann–Liouville delay Hopfield neural network model. The model is defined and initial conditions are set up. The equilibrium is defined in an appropriate way. It depends significantly on the applied fractional derivative. Quadratic Lyapunov-like functions and the modified Razumikhin method are applied to obtain exponential bounds of the solutions of the model. These bounds are obtained on an interval excluding the initial time point. The convergence of any solution to the equilibrium is studied. An example illustrating theoretical results is elaborated in the next section. Some concluding comments are stated in the last section.

## 2. Some Basic Definitions and Preliminaries

The most classical fractional integrals and derivatives as well as their basic properties are well presented in the the classical books; see, for example [18,19,20]. In the last decades, many generalizations of these fractional derivatives have been proposed. Some of them are equivalent to the classical ones, whereas others are generalizations. One of the methods is the exponential factor included in the kernel of the derivative and the integral, and they are called tempered fractional integral and derivative (see, for example, their applications to stochastic process [21]).

Recall that the pair of functions M,K (Sonine kernels) satisfies the relation

(1) $$\left(M*K\right)\left(t\right)=\int_{0}^{t}K\left(s\right)M\left(t-s\right)ds=\left\{1\right\},\;\;t>0,$$

where {1} is the function that is identically equal to 1 for t≥0 (for more details, see [16,17]).

If the functions (M,K) are Sonine kernels then the generalized fractional integral is defined by

(2) $$I_{\left(M\right)}^{RL}f=\int^{0}M\left(t-s\right)f\left(s\right)ds$$

and the generalized fractional derivative of Riemann–Liouville type is defined by

(3) $$D_{\left(K\right)}^{RL}f=\frac{d}{dt}\int^{0}K\left(t-s\right)f\left(s\right)ds.$$

Consider the following pair of functions:

(4) $$M\left(t\right)=\frac{{\left(\lambda t\right)}^{\alpha -1}}{\Gamma (\alpha )}e^{-|\lambda -1|\;t}=\frac{\lambda^{\alpha -1}}{\Gamma (\alpha )}t^{\alpha -1}e^{-|\lambda -1|\;t}$$

and

(5) $$K\left(t\right)=\frac{\lambda^{1-\alpha }}{\Gamma (1-\alpha )}\left(t^{-\alpha }e^{-|\lambda -1|t}+\left|\lambda -1\right|\int_{0}^{t}u^{-\alpha }e^{-|\lambda -1|u}du\right)$$

where α∈(0,1), λ>0.

Both functions M,K are Sonine kernels. Indeed, we have

(6) $$\begin{array}{cc} & K\left(t-s\right)=\frac{\lambda^{1-\alpha }}{\Gamma (1-\alpha )}\left({\left(t-s\right)}^{-\alpha }e^{-|\lambda -1|(t-s)}+\left|\lambda -1\right|\int_{0}^{t-s}u^{-\alpha }e^{-|\lambda -1|u}du\right)\\ & =\frac{\lambda^{1-\alpha }}{\Gamma (1-\alpha )}\left({\left(t-s\right)}^{-\alpha }e^{-|\lambda -1|(t-s)}+\left|\lambda -1\right|\int_{s}^{t}{\left(v-s\right)}^{-\alpha }e^{-|\lambda -1|(v-s)}dv\right),\;\;0<s\leq t, \end{array}$$

and applying that $\int_{0}^{t}s^{\alpha -1}{\left(t-s\right)}^{-\alpha }ds=\Gamma \left(\alpha \right)\Gamma \left(1-\alpha \right)$, we obtain

(7) $$\begin{array}{cc} & \int_{0}^{t}M\left(s\right)K\left(t-s\right)ds=\frac{1}{\Gamma (\alpha )\Gamma (1-\alpha )}(\int_{0}^{t}s^{\alpha -1}e^{-|\lambda -1|t}{\left(t-s\right)}^{-\alpha }ds\\ & \;\;\;\;+|\lambda -1|\int_{0}^{t}s^{\alpha -1}\int_{s}^{t}{\left(v-s\right)}^{-\alpha }e^{-|\lambda -1|v}dvds)\\ & =e^{-|\lambda -1|t}+\frac{1}{\Gamma (\alpha )\Gamma (1-\alpha )}\left|\lambda -1\right|\int_{0}^{t}e^{-|\lambda -1|v}\int_{0}^{v}s^{\alpha -1}{\left(v-s\right)}^{-\alpha }ds\;dv\\ & =e^{-|\lambda -1|t}+\left|\lambda -1\right|\int_{0}^{t}e^{-|\lambda -1|v}dv=1,\;\;t>0. \end{array}$$

Also, we obtain

(8) $$\begin{array}{cc} & \frac{d}{dt}\int_{0}^{t}K\left(t-s\right)\nu \left(s\right)ds=\frac{\lambda^{1-\alpha }}{\Gamma (1-\alpha )}\frac{d}{dt}(\int_{0}^{t}[{\left(t-s\right)}^{-\alpha }e^{-|\lambda -1|(t-s)}\\ & \;\;\;\;+|\lambda -1|\int_{s}^{t}{\left(v-s\right)}^{-\alpha }e^{-|\lambda -1|(v-s)}dv]\nu \left(s\right)ds)\\ & =\frac{\lambda^{1-\alpha }}{\Gamma (1-\alpha )}\frac{d}{dt}(\int_{0}^{t}{\left(t-s\right)}^{-\alpha }e^{-|\lambda -1|(t-s)}\nu \left(s\right)ds\\ & \;\;\;\;+|\lambda -1|\int_{0}^{t}\int_{s}^{t}{\left(v-s\right)}^{-\alpha }e^{-|\lambda -1|(v-s)}\nu \left(s\right)dvds)\\ & =\frac{\lambda^{1-\alpha }}{\Gamma (1-\alpha )}\frac{d}{dt}(\int_{0}^{t}{\left(t-s\right)}^{-\alpha }e^{-|\lambda -1|(t-s)}\nu \left(s\right)ds\\ & \;\;\;\;+|\lambda -1|\int_{0}^{t}\int_{0}^{v}{\left(v-s\right)}^{-\alpha }e^{-|\lambda -1|(v-s)}\nu \left(s\right)dsdv)\\ & =\frac{\lambda^{1-\alpha }}{\Gamma (1-\alpha )}(\frac{d}{dt}\int_{0}^{t}{\left(t-s\right)}^{-\alpha }e^{-|\lambda -1|(t-s)}\nu \left(s\right)ds\\ & \;\;\;\;+|\lambda -1|\int_{0}^{t}{\left(t-s\right)}^{-\alpha }e^{-|\lambda -1|(t-s)}\nu \left(s\right)ds),\;t>0. \end{array}$$

Now, we will define the generalized fractional integral (GFI) and generalized Riemann–Liouville fractional derivative (GRLFD) applying the Sonine kernels M,K given by (4) and (5) and using Equation (8).

**Definition 1.**  *The generalized fractional integral (GFI) of a function υ:[0,A]→R,A≤∞, and λ>0, α≥0, is defined by*

(9) $${}_{0}I_{t}^{\alpha ,\lambda }\upsilon \left(t\right)=\int_{0}^{t}M\left(t-s\right)\nu \left(s\right)ds=\frac{\lambda^{\alpha -1}}{\Gamma (\alpha )}\int_{0}^{t}{\left(t-s\right)}^{\alpha -1}e^{-|\lambda -1|\;(t-s)}\upsilon \left(s\right)\;ds,\;\;\;\;t\in \left(0,A\right].$$

**Definition 2.**  *The generalized Riemann–Liouville fractional derivative (GRLFD) of a function υ:[0,A]→R,A≤∞, and λ>0, α∈(0,1) is defined by*

(10) $$\begin{array}{cc} {}_{0}^{RL}D_{t}^{\alpha ,\lambda }\upsilon \left(t\right) & =\frac{d}{dt}\int_{0}^{t}K\left(t-s\right)\nu \left(s\right)ds\\ & =\frac{\lambda^{1-\alpha }}{\Gamma (1-\alpha )}(\frac{d}{dt}\int_{0}^{t}{\left(t-s\right)}^{-\alpha }e^{-|\lambda -1|(t-s)}\nu \left(s\right)ds\\ & \;\;\;\;+|\lambda -1|\int_{0}^{t}{\left(t-s\right)}^{-\alpha }e^{-|\lambda -1|(t-s)}\nu \left(s\right)ds),\;\;\;t\in \left(0,A\right]. \end{array}$$

**Remark 1.**  *From Equations (9) and (10) in the partial case λ=1, we obtain the classical Riemann fractional integral (RLFI) and Riemann–Liouville fractional derivative (RLFD) (see, for example, [18,19,20]).*

**Remark 2.**  *The defined and applied above kernels are a partial case of Sonine kernels studied in [17]; therefore, the defined GFI and GRLFD by Definitions 1 and 2, respectively, satisfy the first and the second fundamental theorems (see Theorem 4 and Theorem 5 [17]), i.e.,*

$${}_{0}^{RL}D_{t}^{q,\rho }\left({}_{0}I_{t}^{\alpha ,\lambda }\right)\upsilon \left(t\right)=\upsilon \left(t\right),\;t>0,$$

*and*

$${}_{0}I_{t}^{\alpha ,\lambda }\left({}_{0}^{RL}D_{t}^{q,\rho }\right)\upsilon \left(t\right)=\upsilon \left(t\right),\;t>0.$$

**Remark 3.**  *In [14,15], the authors defined another type of integrals and derivatives with exponential kernels. The above-defined GFI and GRLFD are similar to the ones in the above papers but GFI and GRLFD, defined by Definitions 1 and 2, according to Remark 2, are a part of the fractional calculus (which is not true for the ones defined in [14,15]).*

**Proposition 1.**  *For λ>0, α∈(0,1) we have*

(11) $$\begin{array}{cc} & {}_{0}^{RL}D_{t}^{\alpha ,\lambda }\left(e^{-|\lambda -1|t}t^{\alpha -1}\right)=0,\;\;t>0, \end{array}$$

*and*

(12) $$\begin{array}{cc} & {}_{0}^{RL}D_{t}^{\alpha ,\lambda }\left(1\right)=\frac{\lambda }{\Gamma (1-\alpha )}\left(e^{|\lambda -1|t}{\left(\frac{1}{\lambda t}\right)}^{\alpha }+|\frac{\lambda -1}{\lambda }{|}^{\alpha }\gamma (1-\alpha ,|\lambda -1|t,\right),\;\;t>0. \end{array}$$

**Proof.**  From Definition 2 we obtain

(13) $$\begin{array}{cc} & {}_{0}^{RL}D_{t}^{\alpha ,\lambda }\left(e^{-|\lambda -1|t}t^{\alpha -1}\right)=\frac{\lambda^{1-\alpha }}{\Gamma (1-\alpha )}(\frac{d}{dt}\int_{0}^{t}{\left(t-s\right)}^{-\alpha }e^{-|\lambda -1|(t-s)}e^{-|\lambda -1|s}s^{\alpha -1}ds\\ & \;\;\;\;+|\lambda -1|\int_{0}^{t}{\left(t-s\right)}^{-\alpha }e^{-|\lambda -1|(t-s)}e^{-|\lambda -1|s}s^{\alpha -1}ds)\\ & =\frac{\lambda^{1-\alpha }}{\Gamma (1-\alpha )}\left(\frac{d}{dt}e^{-|\lambda -1|t}\int_{0}^{t}{\left(t-s\right)}^{-\alpha }s^{\alpha -1}ds+\left|\lambda -1\right|e^{-|\lambda -1|t}\int_{0}^{t}{\left(t-s\right)}^{-\alpha }s^{\alpha -1}ds\right)\\ & =\frac{\lambda^{1-\alpha }}{\Gamma (1-\alpha )}\left(\frac{d}{dt}e^{-|\lambda -1|t}\Gamma \left(\alpha \right)\Gamma \left(1-\alpha \right)+\left|\lambda -1\right|e^{-|\lambda -1|t}\Gamma \left(\alpha \right)\Gamma \left(1-\alpha \right)\right)=0,\;\;\;t\in \left(0,A\right]. \end{array}$$

We use the equalities

(14) $$\int_{a}^{T}e^{\left|\lambda -1\right|\left(T-s\right)}{\left(T-s\right)}^{-\alpha }ds={\left|\lambda -1\right|}^{\alpha -1}\gamma \left(1-\alpha ,|\lambda -1|\left(T-a\right)\right),\;\;0\leq a\leq T\leq \infty ,$$

with the incomplete lower gamma function γ(β,z),

(15) $$\;\frac{d}{dT}\int_{a}^{T}e^{\left|\lambda -1\right|\left(T-s\right)}{\left(T-s\right)}^{-\alpha }ds=e^{\left|\lambda -1|(T-a\right)}{\left(T-a\right)}^{-\alpha }\;\;0\leq a\leq T\leq \infty ,$$

and we obtain

(16) $$\begin{array}{cc} & {}_{0}^{RL}D_{t}^{\alpha ,\lambda }\left(1\right)=\frac{\lambda^{1-\alpha }}{\Gamma (1-\alpha )}\left(\frac{d}{dt}\int_{0}^{t}{\left(t-s\right)}^{-\alpha }e^{-|\lambda -1|(t-s)}ds+\left|\lambda -1\right|\int_{0}^{t}{\left(t-s\right)}^{-\alpha }e^{-|\lambda -1|(t-s)}ds\right)\\ & =\frac{\lambda^{1-\alpha }}{\Gamma (1-\alpha )}\left(e^{|\lambda -1|t}t^{-\alpha }{+|\lambda -1|}^{\alpha }\gamma (1-\alpha ,|\lambda -1|t,\right),\;\;\;t\in \left(0,A\right]. \end{array}$$

□

## 3. Inequalities for Generalized Proportional Riemann–Liouville Fractional Derivatives

We will use the following class of multivariable functions:

$$\begin{array}{cc} \Omega & =\{V\in C^{2}\left(R^{n},R\right):\;V\left(0\right)=0,\;V\left(\gamma x+\left(1-\gamma \right)y\right)\leq \gamma V\left(x\right)+\left(1-\gamma \right)V\left(y\right)\\ & \;\;\;\;\;\;for\;\gamma \in \left[0,1\right],\;x,y\in R^{n}\}. \end{array}$$

**Remark 4.**  *The function V∈Ω iff $V\in C^{2}\left(R^{n},R\right)$ and $V\left(y\right)\geq V\left(x\right)+\sum_{i=1}^{n}\frac{\partial V(x)}{\partial x_{i}}\left(y_{i}-x_{i}\right)$ for all $x,y\in R^{n},x=\left(x_{1},x_{2},\ldots ,x_{n}\right),\;y=\left(y_{1},y_{2},\ldots ,y_{n}\right)$.*

Define the set of functions:

$$C_{\alpha ,\lambda }\left(\left[0,A\right],R^{n}\right)=\left\{\upsilon :\left[0,A\right]\to R^{n}:\;\forall t\in \left(0,A\right]\;\exists \;\;{}_{0}^{RL}D_{t}^{\alpha ,\lambda }\upsilon \left(t\right)<\infty \right\}.$$

**Lemma 1.**  *Suppose the functions V∈Ω, $x\in C_{\alpha ,\lambda }\left(\left[0,b\right],R^{n}\right),\;b\leq \infty$, $x=(x_{1},x_{2},\ldots ,x_{n}),$ and $V\left(x\left(.\right)\right)\in C_{q,\rho }\left(\left[t_{0},b\right],\left[0,\infty \right)\right)$. Then, the inequality*

(17) $$\left({}_{t_{0}}^{RL}D_{t}^{q,\rho }V(x\left(t\right)\right)\leq \sum_{k=1}^{n}\left({}_{t_{0}}^{RL}D_{t}^{q,\rho }x_{k}\left(t\right)\right)\;\frac{\partial V(x(t))}{\partial x_{k}},\;\;\;t\in \left(0,b\right]$$

*holds.*

**Proof.**  Fix an arbitrary point T∈(0,b]. The inequality (17) is equivalent to

(18) $${}_{0}^{RL}D_{t}^{q,\rho }V\left(y\left(t\right)\right){{|}_{t=T}-\sum_{k=1}^{n}\left({}_{0}^{RL}D_{t}^{q,\rho }y_{k}\left(t\right)\right)|}_{t=T}\;\frac{\partial V(y(T))}{\partial y_{k}}\leq 0.$$

We have

(19) $$x_{k}\left(s\right)=x_{k}\left(0\right)+\int_{0}^{s}\frac{d}{d\xi }x_{k}\left(\xi \right)d\xi ,\;\;k=1,2,\ldots ,n,\;\;s\in \left[0,T\right],$$

and

(20) $$V\left(x\left(s\right)\right)=V\left(x\left(0\right)\right)+\sum_{k=1}^{n}\int_{0}^{s}\frac{\partial V(x(\xi ))}{\partial x_{k}}x_{k}^{\prime }\left(\xi \right)d\xi ,\;\;s\in \left[0,T\right].$$

From Definition 2 and equalities (19) and (20), we obtain

(21) $$\begin{array}{cc} & \frac{\Gamma (1-\alpha )}{\lambda^{1-\alpha }}\left({}_{0}^{RL}D_{t}^{q,\rho }V\left(x\left(t\right)\right){{|}_{t=T}-\sum_{k=1}^{n}\frac{\partial V(x(T))}{\partial x_{k}}{}_{0}^{RL}D_{t}^{q,\rho }x_{k}\left(t\right)|}_{t=T}\right)\\ & =\frac{d}{dT}\int_{0}^{T}{\left(T-s\right)}^{-\alpha }e^{-|\lambda -1|(T-s)}\left(V\left(x\left(0\right)\right)+\sum_{k=1}^{n}\int_{0}^{s}\frac{\partial V(x(\xi ))}{\partial x_{k}}x_{k}^{\prime }\left(\xi \right)d\xi \right)ds\\ & \;\;\;\;+|\lambda -1|\int_{0}^{T}{\left(T-s\right)}^{-\alpha }e^{-|\lambda -1|(T-s)}\left(V\left(x\left(0\right)\right)+\sum_{k=1}^{n}\int_{0}^{s}\frac{\partial V(x(\xi ))}{\partial x_{k}}x_{k}^{\prime }\left(\xi \right)d\xi \right)ds\\ & \;\;\;\;-\sum_{k=1}^{n}\frac{\partial V(x(T))}{\partial x_{k}}\frac{d}{dT}\int_{0}^{T}{\left(T-s\right)}^{-\alpha }e^{-|\lambda -1|(T-s)}\left(x_{k}\left(0\right)+\int_{0}^{s}x_{k}^{\prime }\left(\xi \right)d\xi \right)ds\\ & \;\;\;\;-|\lambda -1|\sum_{k=1}^{n}\frac{\partial V(x(T))}{\partial x_{k}}\int_{0}^{T}{\left(T-s\right)}^{-\alpha }e^{-|\lambda -1|(T-s)}\left(x_{k}\left(0\right)+\int_{0}^{s}x_{k}^{\prime }\left(\xi \right)d\xi \right)ds\\ & =\left(V\left(x\left(0\right)\right)-\frac{\partial V(x(T))}{\partial x_{k}}x_{k}\left(0\right)\right)\frac{d}{dT}\int_{0}^{T}{\left(T-s\right)}^{-\alpha }e^{-|\lambda -1|(T-s)}ds\\ & \;\;\;\;+\frac{d}{dT}\int_{0}^{T}{\left(T-s\right)}^{-\alpha }e^{-|\lambda -1|(T-s)}\sum_{k=1}^{n}\int_{0}^{s}\frac{\partial V(x(\xi ))}{\partial x_{k}}x_{k}^{\prime }\left(\xi \right)d\xi ds\\ & \;\;\;\;-\sum_{k=1}^{n}\frac{\partial V(x(T))}{\partial x_{k}}\frac{d}{dT}\int_{0}^{T}{\left(T-s\right)}^{-\alpha }e^{-|\lambda -1|(T-s)})\int_{0}^{s}x_{k}^{\prime }\left(\xi \right)d\xi ds\\ & \;\;\;\;\;\;+|\lambda -1|\left(V\left(x\left(0\right)\right)-\frac{\partial V(x(T))}{\partial x_{k}}x_{k}\left(0\right)\right)\int_{0}^{T}{\left(T-s\right)}^{-\alpha }e^{-|\lambda -1|(T-s)}ds\\ & \;\;\;\;+|\lambda -1|\int_{0}^{T}{\left(T-s\right)}^{-\alpha }e^{-|\lambda -1|(T-s)}\sum_{k=1}^{n}\int_{0}^{s}\frac{\partial V(x(\xi ))}{\partial x_{k}}x_{k}^{\prime }\left(\xi \right)d\xi ds\\ & \;\;\;\;-|\lambda -1|\sum_{k=1}^{n}\frac{\partial V(x(T))}{\partial x_{k}}\int_{0}^{T}{\left(T-s\right)}^{-\alpha }e^{-|\lambda -1|(T-s)}\int_{0}^{s}x_{k}^{\prime }\left(\xi \right)d\xi ds. \end{array}$$

We apply (19), (20), (14), (15), and the equalities

$$\int_{0}^{T}\int_{0}^{s}f\left(s,\xi \right)d\xi ds=\int_{0}^{T}\int_{\xi }^{T}f\left(s,\xi \right)dsd\xi$$

for $f\left(s,\xi \right)=e^{-|\lambda -1|(T-s)}{\left(T-s\right)}^{-\alpha }\frac{\partial V(x(\xi ))}{\partial x_{k}}x_{k}^{\prime }\left(\xi \right)$ or $f\left(s,\xi \right)=e^{-|\lambda -1|(T-s)}{\left(T-s\right)}^{-\alpha }x_{k}^{\prime }\left(\xi \right)$ to equality (21) and we obtain

(22) $$\begin{array}{cc} & \frac{\Gamma (1-\alpha )}{\lambda^{1-\alpha }}\left({}_{0}^{RL}D_{t}^{q,\rho }V\left(x\left(t\right)\right){{|}_{t=T}-\sum_{k=1}^{n}\frac{\partial V(x(T))}{\partial x_{k}}{}_{0}^{RL}D_{t}^{q,\rho }x_{k}\left(t\right)|}_{t=T}\right)\\ & =\left(V\left(x\left(0\right)\right)-\frac{\partial V(x(T))}{\partial x_{k}}x_{k}\left(0\right)\right)e^{-|\lambda -1|T}T^{-\alpha }\\ & \;\;\;\;+\sum_{k=1}^{n}\frac{d}{dT}\int_{0}^{T}\int_{\xi }^{T}{\left(T-s\right)}^{-\alpha }e^{-|\lambda -1|(T-s)}\frac{\partial V(x(\xi ))}{\partial x_{k}}x_{k}^{\prime }\left(\xi \right)dsd\xi \\ & \;\;\;\;-\sum_{k=1}^{n}\frac{\partial V(x(T))}{\partial x_{k}}\frac{d}{dT}\int_{0}^{T}\int_{\xi }^{T}{\left(T-s\right)}^{-\alpha }e^{-|\lambda -1|(T-s)})x_{k}^{\prime }\left(\xi \right)dsd\xi \\ & \;\;\;\;\;\;+|\lambda -1|\left(V\left(x\left(0\right)\right)-\frac{\partial V(x(T))}{\partial x_{k}}x_{k}\left(0\right)\right)\int_{0}^{T}{\left(T-s\right)}^{-\alpha }e^{-|\lambda -1|(T-s)}ds\\ & \;\;\;\;+|\lambda -1|\int_{0}^{T}\sum_{k=1}^{n}\left(\frac{\partial V(x(\xi ))}{\partial x_{k}}-\frac{\partial V(x(T))}{\partial x_{k}}\right)x_{k}^{\prime }\left(\xi \right)\int_{\xi }^{T}{\left(T-s\right)}^{-\alpha }e^{-|\lambda -1|(T-s)}dsd\xi . \end{array}$$

Note that we have

(23) $$\begin{array}{cc} & \frac{d}{dT}\int_{0}^{T}\int_{\xi }^{T}\frac{\partial V(x(\xi ))}{\partial x_{k}}x_{k}^{\prime }\left(\xi \right)e^{-|\lambda -1|\left(T-s\right)}{\left(T-s\right)}^{-\alpha }ds\;d\xi \\ & =\int_{0}^{T}\frac{\partial V(x(\xi ))}{\partial x_{k}}x_{k}^{\prime }\left(\xi \right)\frac{d}{dT}\int_{\xi }^{T}e^{-|\lambda -1|\left(T-s\right)}{\left(T-s\right)}^{-\alpha }ds\;d\xi \\ & =\int_{0}^{T}\frac{\partial V(x(\xi ))}{\partial x_{k}}x_{k}^{\prime }\left(\xi \right)e^{-|\lambda -1|(T-\xi )}{\left(T-\xi \right)}^{-\alpha }\;d\xi \end{array}$$

and

(24) $$\begin{array}{cc} & \frac{d}{dT}\int_{0}^{T}x_{k}^{\prime }\left(\xi \right)\int_{\xi }^{T}e^{-|\lambda -1|\left(T-s\right)}{\left(T-s\right)}^{-\alpha }ds\;d\xi =\int_{0}^{T}x_{k}^{\prime }\left(\xi \right)e^{-|\lambda -1|(T-\xi )}{\left(T-\xi \right)}^{-\alpha }\;d\xi . \end{array}$$

We substitute (23) and (24) in (22) and we obtain

(25) $$\begin{array}{cc} & \frac{\Gamma (1-\alpha )}{\lambda^{1-\alpha }}\left({}_{0}^{RL}D_{t}^{q,\rho }V\left(x\left(t\right)\right){{|}_{t=T}-\sum_{k=1}^{n}\frac{\partial V(x(T))}{\partial x_{k}}{}_{0}^{RL}D_{t}^{q,\rho }x_{k}\left(t\right)|}_{t=T}\right)\\ & =\left(V\left(x\left(0\right)\right)-\frac{\partial V(x(T))}{\partial x_{k}}x_{k}\left(0\right)\right)e^{-|\lambda -1|T}T^{-\alpha }\\ & \;\;\;\;+\int_{0}^{T}\sum_{k=1}^{n}\left(\frac{\partial V(x(\xi ))}{\partial x_{k}}-\frac{\partial V(x(T))}{\partial x_{k}}\right)x_{k}^{\prime }\left(\xi \right)e^{-|\lambda -1|(T-\xi )}{\left(T-\xi \right)}^{-\alpha }\;d\xi \\ & \;\;\;\;\;\;+|\lambda -1|\left(V\left(x\left(0\right)\right)-\frac{\partial V(x(T))}{\partial x_{k}}x_{k}\left(0\right)\right)\int_{0}^{T}{\left(T-s\right)}^{-\alpha }e^{-(\lambda -1)(T-s)}ds\\ & \;\;\;\;+|\lambda -1|\int_{0}^{T}\sum_{k=1}^{n}\left(\frac{\partial V(x(\xi ))}{\partial x_{k}}-\frac{\partial V(x(T))}{\partial x_{k}}\right)x_{k}^{\prime }\left(\xi \right)\int_{\xi }^{T}{\left(T-s\right)}^{-\alpha }e^{-|\lambda -1|(T-s)}dsd\xi . \end{array}$$

We define the function $P\left(s\right)=V\left(x\left(s\right)\right)-V\left(x\left(T\right)\right)-\sum_{k=1}^{n}\frac{\partial V(x(T))}{\partial x_{k}})\left[x_{k}\left(s\right)-x_{k}\left(T\right)\right]$ for s∈[0,T]. From V∈Ω it follows that P(s)≥0 for all s∈[0,T], P(T)=0 and $\frac{dP(s)}{ds}=\sum_{k=1}^{n}\left(\frac{\partial V(x(s))}{\partial x_{k}}-\frac{\partial V(x(T))}{\partial x_{k}}\right)x_{k}^{\prime }\left(s\right)$.

We integrate by parts and use the equalities $\lim_{s\to T-}\frac{P(s)}{{\left(T-s\right)}^{q}}=\lim_{s\to T-}\frac{P^{\prime \prime }\left(s\right)}{q(q-1)}{\left(T-s\right)}^{2-\alpha }=0$ and $\frac{d}{ds}\left(e^{-|\lambda -1|(T-s)}{\left(T-s\right)}^{-\alpha }\right)=e^{\left|\lambda -1|(T-s\right)}{\left(T-s\right)}^{-\alpha }{(-|\lambda -1|+\alpha \left(T-s\right)}^{-1})$ and we obtain

(26) $$\begin{array}{cc} & \int_{0}^{T}P^{\prime }\left(\xi \right)\int_{\xi }^{T}{\left(T-s\right)}^{-\alpha }e^{-|\lambda -1|(T-s)}dsd\xi \\ & =P\left(\xi \right)\int_{\xi }^{T}{\left(T-s\right)}^{-\alpha }e^{-|\lambda -1|(T-s)}ds{|}_{\xi =0}^{\xi =T}-\int_{0}^{T}P\left(\xi \right)\frac{d}{d\xi }\int_{\xi }^{T}{\left(T-s\right)}^{-\alpha }e^{-|\lambda -1|(T-s)}ds\\ & =-P\left(0\right)\int_{0}^{T}{\left(T-s\right)}^{-\alpha }e^{-|\lambda -1|(T-s)}ds-\int_{0}^{T}P\left(\xi \right){\left(T-\xi \right)}^{-\alpha }e^{-|\lambda -1|(T-\xi )}d\xi \\ & \leq -P\left(0\right)\int_{0}^{T}{\left(T-s\right)}^{-\alpha }e^{-|\lambda -1|(T-s)}ds, \end{array}$$

and

(27) $$\begin{array}{cc} & \int_{0}^{T}P^{\prime }\left(\xi \right){\left(T-\xi \right)}^{-\alpha }e^{-|\lambda -1|(T-\xi )}d\xi \\ & =P\left(\xi \right){\left(T-\xi \right)}^{-\alpha }e^{-|\lambda -1|(T-\xi )}d\xi {|}_{\xi =0}^{\xi =T}-\int_{0}^{T}P\left(\xi \right)\frac{d}{d\xi }{\left(T-\xi \right)}^{-\alpha }e^{-|\lambda -1|(T-\xi )}\\ & =-P\left(0\right)T^{-\alpha }e^{-|\lambda -1|T}\\ & \;-\int_{0}^{T}P\left(\xi \right)\left(\alpha {\left(T-\xi \right)}^{\alpha -1}e^{-|\lambda -1|(T-\xi )}+\left|\lambda -1\right|{\left(T-\xi \right)}^{-\alpha }e^{-|\lambda -1|(T-\xi )}\right)d\xi \\ & \leq -P\left(0\right)T^{-\alpha }e^{-|\lambda -1|T}ds. \end{array}$$

From V∈Ω and Remark 4 with y=0, it follows that $-V\left(x\left(T\right)\right)+\sum_{k=1}^{n}\frac{\partial V(x(T))}{\partial x_{k}}x_{k}\left(T\right)\geq 0$; thus, from (25), (27), and (26) we obtain

(28) $$\begin{array}{cc} & \frac{\Gamma (1-\alpha )}{\lambda^{1-\alpha }}\left({}_{0}^{RL}D_{t}^{q,\rho }V\left(x\left(t\right)\right){{|}_{t=T}-\sum_{k=1}^{n}\frac{\partial V(x(T))}{\partial x_{k}}{}_{0}^{RL}D_{t}^{q,\rho }x_{k}\left(t\right)|}_{t=T}\right)\\ & =P\left(0\right)e^{-|\lambda -1|T}T^{-\alpha }-\left(-V\left(x\left(T\right)\right)+\sum_{k=1}^{n}\frac{\partial V(x(T))}{\partial x_{k}}x_{k}\left(T\right)\right)e^{-|\lambda -1|T}T^{-\alpha }\\ & \;\;\;\;+\int_{0}^{T}P^{\prime }\left(\xi \right)e^{-|\lambda -1|(T-\xi )}{\left(T-\xi \right)}^{-\alpha }\;d\xi \\ & \;\;\;\;\;\;+|\lambda -1|P\left(0\right)\int_{0}^{T}{\left(T-s\right)}^{-\alpha }e^{-(\lambda -1)(T-s)}ds\\ & \;\;\;\;\;\;+|\lambda -1|\left(-V\left(x\left(T\right)\right)-\frac{\partial V(x(T))}{\partial x_{k}}x_{k}\left(T\right)\right)\int_{0}^{T}{\left(T-s\right)}^{-\alpha }e^{-(\lambda -1)(T-s)}ds\\ & \;\;\;\;+|\lambda -1|\int_{0}^{T}P^{\prime }\left(\xi \right)\int_{\xi }^{T}{\left(T-s\right)}^{-\alpha }e^{-|\lambda -1|(T-s)}dsd\xi \\ & \leq P\left(0\right)e^{-|\lambda -1|T}T^{-\alpha }-\left(-V\left(x\left(T\right)\right)+\sum_{k=1}^{n}\frac{\partial V(x(T))}{\partial x_{k}}x_{k}\left(T\right)\right)e^{-|\lambda -1|T}T^{-\alpha }\\ & \;\;\;\;-P\left(0\right)e^{-|\lambda -1|T}T^{-\alpha }+\left|\lambda -1\right|P\left(0\right)\int_{0}^{T}{\left(T-s\right)}^{-\alpha }e^{-(\lambda -1)(T-s)}ds\\ & \;\;\;\;\;\;-|\lambda -1|\left(-V\left(x\left(T\right)\right)+\frac{\partial V(x(T))}{\partial x_{k}}x_{k}\left(T\right)\right)\int_{0}^{T}{\left(T-s\right)}^{-\alpha }e^{-(\lambda -1)(T-s)}ds\\ & \;\;\;\;-|\lambda -1|P\left(0\right)\int_{0}^{T}{\left(T-s\right)}^{-\alpha }e^{-|\lambda -1|(T-s)}ds\leq 0. \end{array}$$

□

In the case $V\left(x\right)=\sum_{k=1}^{n}x_{k}^{2}$ for $x\in R^{n}:\;x=\left(x_{1},x_{2},dots,x_{n}\right)$, we obtain as a partial case of Lemma 1 the following result.

**Corollary 1.**  *Let the function $\nu \in C_{\alpha ,\lambda }\left(\left[0,A\right],R^{n}\right)$, 0<A≤∞, and $\nu^{T}\nu \in C_{\alpha ,\lambda }\left(\left[0,A\right],R\right)$. Then, the inequality ${}_{0}^{RL}D_{t}^{\alpha ,\lambda }\nu^{T}\nu \left(t\right)\leq 2\nu^{T}\left(t\right){}_{0}^{RL}D_{t}^{\alpha ,\lambda }\nu \left(t\right)$ for t∈(0,A] holds.*

In our further study we will use the following result, for which the proof is very similar to the one in [22] and we omit it.

**Lemma 2.**  *Let the function υ∈C([0,A],R), 0<A<∞ be Lipshitz, and there exists a point T∈(0,A] such that υ(T)=0, and υ(t)<0, for 0≤t<T. Then, if the GRLFD of υ exists for t=T with α∈(0,1),λ>0, then the inequality $\left({\;}_{0}^{RL}D^{\alpha ,\lambda }\upsilon \right)\left(t\right){|}_{t=T}\geq 0$ holds.*

**Remark 5.**  *Note that a similar result to the one in Lemma 2 is proved in [23] for Riemann–Liouville fractional derivatives.*

## 4. Some Results for Delay Differential Equations with GRLFD

Consider the delay nonlinear differential equation with GRLFD

(29) $$\begin{array}{cc} & {}_{0}^{RL}D_{t}^{\alpha ,\lambda }y\left(t\right)=f\left(t,y_{t}\right)\;\;for\;t>0, \end{array}$$

with initial conditions

(30) $$\begin{array}{cc} & y(t)=\phi (t),\;for\;t\in [-\tau ,0),\\ & {\;}_{0}I_{t}^{1-\alpha ,\lambda }{y\left(t\right)|}_{t=0+}=\phi \left(0\right), \end{array}$$

where α∈(0,1),λ>0, $y_{t}\left(\sigma \right)=y\left(t+\sigma \right)$ for σ∈[−τ,0], the initial function $\phi :\left[-\tau ,0\right]\to R^{n}$ and $f:R_{+}\times R^{n}\to R^{n}$.

We will assume that the initial value problem problem (29), (30) has a solution $y\left(t;\phi \right)\in C_{\alpha ,\lambda }\left(\left[0,\infty \right),R^{n}\right)$ for any initial function $\phi \in C(\left[-\tau ,0\right],R^{n}))$.

For any vector $\xi =(\xi_{1},\xi_{2},\ldots ,\xi_{n})$, we will use the norm $\left||\xi |\right|=\sqrt{\sum_{i=1}^{n}\xi_{i}^{2}}$.

We will use the norm in $C(\left[-\tau ,0\right],R^{n})$, defined by ${\left||\phi |\right|}_{0}=\max_{t\in [-\tau ,0]}\left||\phi \left(t\right)|\right|$.

**Theorem 1.**  *Let there exist a continuous locally Lipschitz function $V:R^{n}\to \left[0,\infty \right)$ such that*

*(i)*  *There exists an increasing function a∈C([0,∞),[0,∞)):a(0)=0, such that a(||ξ||)≤V(ξ) for $\xi \in R^{n};$*
*(ii)*  *For any solution $y\in C_{\alpha ,\lambda }\left(\left[0,\infty \right),R^{n}\right)$ of (29), (30), the following conditions are satisfied:*
  - *There exists an increasing function g∈C([0,∞),R):g(0)=0 such that the inequality*
$$\lim_{t\to 0+}e^{-|\lambda -1|t}t^{1-\alpha }V\left(y\left(t\right)\right)\leq g\left(||\phi \left(0\right)||\right)$$
    *holds;*
  - *For any point, T>0 such that*
$$e^{-|\lambda -1|(T+\sigma )}{\left(T+\sigma \right)}^{1-\alpha }V\left(y\left(T+\sigma \right)\right)<e^{-|\lambda -1|t}T^{1-\alpha }V\left(y\left(T\right)\right)for\;\;\sigma \in \left(-\min\left\{T,\tau \right\},0\right),$$
    *the GRLFD ${\;}_{0}^{RL}D_{t}^{\alpha ,\lambda }V\left(y\left(t\right)\right){|}_{t=T}$ exists, and the inequality*
$${\;}_{0}^{RL}D_{t}^{\alpha ,\lambda }V\left(y\left(t\right)\right){|}_{t=T}<0$$
    *holds.*

*Then, there exists a point $T_{\alpha }>0$ such that for any solution of (29), (30), the inequality*

$$||x\left(t\right)||<a^{-1}\left({g(||\phi ||}_{0})e^{-|\lambda -1|t}\right)\;\;for\;t>T_{\alpha }$$

*holds.*

**Proof.**  Let x(t) be a solution of (29), (30) with the initial function $\phi \in C(\left[-\tau ,0\right],R^{n})$.

From condition (ii), we obtain

(31) $$\begin{array}{cc} & \lim_{\sigma \to 0+}e^{-|\lambda -1|\sigma }\sigma^{1-\alpha }V\left(x\left(\sigma \right)\right)\leq g\left(||\phi (0)||\right)\leq {g(||\phi ||}_{0}). \end{array}$$

Therefore, there exists a number δ>0 such that

(32) $$V\left(x\left(t\right)\right)\leq g\left({\left||\phi |\right|}_{0}\right)<e^{-|\lambda -1|t}t^{\alpha -1}{g(||\phi ||}_{0}),\;\;for\;t\in \left(0,\delta \right).$$

We define the function $H\left(t\right)={g(||\phi ||}_{0})e^{-|\lambda -1|t}t^{\alpha -1}\in C_{\alpha ,\lambda }\left(\left[0,\infty \right),R_{+}\right)$ and $\lim_{t\to \infty }H\left(t\right)$ = 0. Then, there exists $T_{\alpha }>0$ such that $t^{\alpha -1}<1$ for $\;t>T_{\alpha }$, and, thus,

(33) $${H\left(t\right)<g(||\phi ||}_{0})e^{-|\lambda -1|t}\;\;for\;t>T_{\alpha }.$$

Consider the function $m\left(t\right)=V\left(x\left(t\right)\right)\in C_{\alpha ,\lambda }\left(\left[0,\infty \right),R_{+}\right)$.

We will prove that

(34) m(t)<H(t),t>0.

Assume inequality (34) is not true for all t>0. Then, there exists a point η≥δ>0 such that

(35) m(η)=H(η),andm(t)<H(t),t∈(0,η).

Thus, $m\left(t\right)-H\left(t\right)\in C_{\alpha ,\lambda }\left(\left[0,\eta \right],R\right)$. According to Lemma 2 with T=η,ν(t)≡m(t)−H(t), the inequality ${\;}_{0}^{RL}D_{t}^{\alpha ,\lambda }\left(m(t)-H(t)\right){|}_{t=\eta }\geq 0$ holds. From Proposition 1, we obtain ${}_{0}^{RL}D^{\alpha ,\lambda }\left(e^{-|\lambda -1|t}t^{\alpha -1}\right)=0$ and, therefore,

(36) $${\;}_{0}^{RL}D_{t}^{\alpha ,\lambda }m\left(t\right){{|}_{t=\eta }={\;}_{0}^{RL}D_{t}^{\alpha ,\lambda }\left(m(t)-H(t)\right)|}_{t=\eta }\geq 0.$$

*Case 1.* Let η>τ. Then, min{η,τ}=τ. From (35), it follows that

$$e^{-|\lambda -1|t}t^{1-\alpha }m\left(t\right)<\varepsilon =e^{-|\lambda -1|\eta }\eta^{1-\alpha }m\left(\eta \right),\;\;\;\;t\in \left(0,\eta \right)$$

or

$$e^{-|\lambda -1|(\eta +\sigma )}{\left(\eta +\sigma \right)}^{1-\alpha }V\left(\xi +\sigma ,x\left(\eta +\sigma \right)\right)<e^{-|\lambda -1|\eta }\eta^{1-\alpha }V\left(\eta ,x\left(\eta \right)\right),\;\;\;\;\sigma \in \left(-\tau ,0\right).$$

According to condition 2(iii), the inequality

(37) $${\;}_{0}^{RL}D_{t}^{\alpha ,\lambda }{V\left(t,x\left(t\right)\right)|}_{t=\eta }<0$$

holds.

The inequality (37) contradicts (36).

*Case 2.* Let η≤τ. Then, min{η,τ}=ξ. From (35), it follows that

$$e^{-|\lambda -1|\eta }\eta^{1-\alpha }m\left(\eta \right)=\varepsilon >e^{-|\lambda -1|t}t^{1-\alpha }m\left(t\right),\;\;\;\;t\in \left(0,\eta \right),$$

or

$$e^{-|\lambda -1|(\eta +\sigma )}{\left(\eta +\sigma \right)}^{1-\alpha }V\left(\eta +\sigma ,x\left(\eta +\sigma \right)\right)<e^{-|\lambda -1|\eta }\eta^{1-\alpha }m\left(\eta \right)=e^{-|\lambda -1|\eta }\eta^{1-\alpha }V\left(\eta ,x\left(\eta \right)\right)$$

for σ∈(−ξ,0). Similar to Case 1, we obtain a contradiction.

From inequalities (33), (34) and condition (i), it follows that

(38) $$a\left(||x\left(t\right)||\right)\leq V\left(x\left(t\right)\right)={m\left(t\right)<H\left(t\right)<g(||\phi ||}_{0})e^{-|\lambda -1|t}\;\;for\;t>T_{\alpha }.$$

It proves the claim of Theorem 1. □

Using that for any fixed T≥0 the function $Q\left(\sigma \right)=e^{-|\lambda -1|(T+\sigma )}{\left(T+\sigma \right)}^{1-\alpha }$ is an increasing function for σ∈(−min{T,τ},0) we obtain the following result.

**Corollary 2.**  *Assume the condition (i) of Theorem 1:*

(ii)${}^{*}$ *For any solution $y\in C_{\alpha ,\lambda }\left(\left[0,\infty \right),R^{n}\right)$ of (29), (30), the following conditions are satisfied:*
  - *There exists an increasing function g∈C([0,∞),R):g(0)=0 such that the inequality*
$$e^{-|\lambda -1|t}t^{1-\alpha }{V\left(y\left(t\right)\right)|}_{t=0+}=\lim_{t\to 0+}e^{-|\lambda -1|t}t^{1-q}V\left(y\left(t\right)\right)\leq g\left(||\phi \left(0\right)||\right)$$
    *holds;*
  - *For any point T>0 such that*
V(y(T+σ))<V(y(T))forσ∈(−min{T,τ},0),
    *the GRLFD ${\;}_{0}^{RL}D_{t}^{\alpha ,\lambda }V\left(y\left(t\right)\right){|}_{t=T}$ exists and the inequality*
$${\;}_{0}^{RL}D_{t}^{\alpha ,\lambda }V\left(y\left(t\right)\right){|}_{t=T}<0$$
    *holds.*

*Then, any solution of (29), (30) satisfies the inequality*

$$||x\left(t\right)||<a^{-1}\left({g(||\phi ||}_{0})e^{-|\lambda -1|t}\right)\;\;for\;t>T_{\alpha }.$$

In the case that the Lyapunov function is a quadratic one, we obtain the following result.

**Corollary 3.**  *Let, for any solution $y\in C_{\alpha ,\lambda }\left(\left[0,\infty \right),R^{n}\right)$ of (29), (30), $y^{T}y\in C_{\alpha ,\lambda }\left(\left[0,\infty \right),R^{n}\right)$ holds and*

- *There exists an increasing function g∈C([0,∞),R):g(0)=0 such that the inequality*
$$\lim_{t\to 0+}e^{-|\lambda -1|t}t^{1-\alpha }y^{T}\left(t\right)y\left(t\right)\leq {g(||\phi \left(0\right)||}_{2})$$
  *holds;*
- *For any point T>0 such that*
(39) $$y^{T}\left(T+\sigma \right)y\left(T+\sigma \right)<y^{T}\left(T\right)y\left(T\right)\;\;for\;\;\sigma \in \left(-\min\left\{T,\tau \right\},0\right),$$
  *the GRLFD ${\;}_{0}^{RL}D_{t}^{\alpha ,gl}y^{T}\left(t\right)y\left(t\right){|}_{t=T}$ exists and the inequality*
$${\;}_{0}^{RL}D_{t}^{\alpha ,\lambda }y^{T}\left(t\right)y\left(t\right){|}_{t=T}<0$$
  *holds.*

*Then, there exists $T_{\alpha }>0$ such that*

$$||y\left(t\right)||<\sqrt{g\left(\max_{t\in [-\tau ,0]}{\left||\phi |\right|}_{2}\right)}e^{-0.5|\lambda -1|t}\;\;for\;t>T_{\alpha }.$$

The proof follows from Theorem 1 by taking the Lyapunov function $V\left(x\right)=\sum_{i=1}^{n}x_{i}^{2}$ and using $a\left(u\right)\equiv u^{2}$.

## 5. Delayed Model of Neural Networks by GRLFD

### 5.1. Model Description

We will consider the general model of Hopfield neural network with the GRLFD and time-varying delays and distributed delays:

(40) $$\begin{array}{cc} & {}_{0}^{RL}D_{t}^{\alpha ,\lambda }u_{i}\left(t\right)=-A_{i}\left(t\right)u_{i}\left(t\right)+\sum_{k=1}^{n}a_{i,k}\left(t\right)f_{k}\left(u_{k}\left(t\right)\right)\\ & +\sum_{k=1}^{n}b_{i,k}\left(t\right)g_{k}\left(u_{k}\left(t-\kappa \left(t\right)\right)\right)+\sum_{k=1}^{n}c_{i,k}\left(t\right)\int_{t-\Theta (t)}^{t}h_{k}\left(u_{k}\left(s\right)\right)ds+I_{i}\left(t\right),\;\;t>0,\;\;i=1,2,\ldots ,n, \end{array}$$

where $u_{i}\left(t\right)$, i=1,2,…,n are the state variables of the *i*-th neuron at time t>0, $a_{ij}\left(t\right),b_{ij}\left(t\right),c_{ij}\left(t\right)$ represent the strengths of the neuron interconnection at time *t* (assuming they are time changeable), *n* is the number of units in the neural network, α∈(0,1),λ>0, $f_{j}\left(u\right)$, $g_{j}\left(u\right)$ and $h_{j}\left(u\right)$ are the activation functions of the *j*-th neuron, κ(t) is the time-varying delay, and Θ(t) is the length of interval of the distributed delay with 0≤κ(t)≤κ,0≤Θ(t)≤Θ, and τ=max{κ,Θ}, $I_{i}\left(t\right)$ are the external inputs at time *t*.

The initial time interval is [−τ,0]. The applied GRLFD leads to a singularity of the solutions at the initial time 0. It requires appropriate definition of the initial conditions. Some authors (see, for example, [24] for RLFD) used the integral of the type ${}_{0}I_{t}^{1-\alpha }u\left(t\right)$ for t∈[−τ,0] in the initial condition but this integral is defined for *t* greater than the lower limit, which is 0 in our case.

We will use the initial conditions (30).

We use the assumptions:

A1. The function $A_{i}\in C\left(R,\left[\mu_{i},\infty \right)\right)$ where $\mu_{i},\;i=1,2,\ldots ,n,$ are positive constants.
A2. The activation functions $f_{i},g_{i},h_{i}\in C\left(R,R\right)$ are Lipschitz with constants $\alpha_{i},\beta_{i},\gamma_{i},\;i=1,2,\ldots ,n$, respectively, i.e.,
  $$|f_{i}\left(x\right)-f_{i}\left(y\right)|\leq \alpha_{i}\left|x-y|,\;\;\;\;\right|g_{i}\left(x\right)-g_{i}\left(y\right)|\leq \beta_{i}\left|x-y|,\;\;\;\;\right|h_{i}\left(x\right)-h_{i}\left(y\right)|\leq \gamma_{i}\left|x-y\right|,\;\;x,y\in R.$$
A3. The functions $a_{ij},b_{ij},c_{ij}\in C\left(\left[0,\infty \right),R\right)$, i,j=1,2,…,n.

### 5.2. Equilibrium of the Model

We define the equilibrium as a constant vector.

We also need to use the equality (16).

**Definition 3.**  The constant vector $V^{*}=\left(C_{1},C_{2},\ldots ,C_{n}\right)$ is called an equilibrium of (40) if the equalities

(41) $$\begin{array}{cc} & C_{i}\frac{\lambda^{1-\alpha }}{\Gamma (1-\alpha )}\left(e^{|\lambda -1|t}t^{-\alpha }{+|\lambda -1|}^{\alpha }\gamma \left(1-\alpha ,|\lambda -1|t\right)\right)\\ & =-A_{i}\left(t\right)C_{i}+\sum_{k=1}^{n}\left(a_{i,k}\left(t\right)f_{k}\left(C_{k}\right)+b_{i,k}\left(t\right)g_{k}\left(C_{k}\right)+c_{i,k}\left(t\right)\Theta \left(t\right)h_{k}\left(C_{k}\right)\right)+I_{i}\left(t\right)\\ & \;\;\;\;\;\;\;\;for\;\;t\geq 0,\;\;i=1,2\ldots ,n \end{array}$$

*hold.*

**Remark 6.**  The equilibrium $V^{*}$ satisfies the initial condition

$${}_{0}I_{t}^{1-\alpha ,\lambda }C_{k}=\frac{\lambda^{\alpha -1}C_{k}}{\Gamma (\alpha )}\int_{0}^{t}{\left(t-s\right)}^{\alpha -1}e^{-|\lambda -1|\;(t-s)}ds=\frac{\lambda^{\alpha -1}C_{k}}{\Gamma (\alpha )}{\left|\lambda -1\right|}^{\alpha -1}{\gamma (1-\alpha ,|\lambda -1|t)|}_{t=0+}=0.$$

**Remark 7.**  *If $f_{i}\left(0\right)=g_{i}\left(0\right)=h_{i}\left(0\right)=0,\;i=1,2,\ldots ,n$ and there is no external input, then the model (40) has a zero equilibrium.*

**Remark 8.**  *In most cases in the literature, the Hopfield neural networks are studied with constant coefficients $a_{ik},b_{ik},c_{ik}$ and constant external inputs $I_{i}$. In the case when RLFD or GRLFD is applied and all coefficients are constants, the only equilibrium is zero. It is not the case when at least one of the coefficients is variable in time.*

Let $V^{*}$ be an equilibrium of (40). We change the variables $\nu_{i}\left(t\right)=u_{i}\left(t\right)-C_{i},\;t\geq 0$, in system (40). Then, applying (41), we obtain

(42) $$\begin{array}{cc} & {}_{0}^{RL}D_{t}^{q,\rho }\nu_{i}\left(t\right)=-A_{i}\left(t\right)\nu_{i}\left(t\right)+\sum_{k=1}^{n}a_{i,k}\left(t\right)F_{k}\left(\nu_{k}\left(t\right)\right)+\sum_{k=1}^{n}b_{i,k}\left(t\right)G_{k}\left(\nu_{k}\left(t-\xi \left(t\right)\right)\right)\\ & +\sum_{k=1}^{n}c_{i,k}\left(t\right)\int_{t-\Theta (t)}^{t}H_{k}\left(\nu_{k}\left(s\right)\right)ds,\;\;\;t>0,\;\;\;i=1,2,\ldots ,n, \end{array}$$

with initial conditions (30), where

$$F_{i}\left(x\right)=f_{i}\left(x+C_{i}\right)-f_{i}\left(C_{i}\right),\;G_{i}\left(x\right)=g_{i}\left(x+C_{i}\right)-g_{i}\left(C_{i}\right),,\;H_{i}\left(x\right)=h_{i}\left(x+C_{i}\right)-h_{i}\left(C_{i}\right).$$

Note that the system (42) has a zero solution (with zero initial function).

**Remark 9.**  *If assumption A2 is satisfied, then*

$$|F_{i}\left(x\right)|\leq \alpha_{i}\left|x|,\;\;\;\;\right|G_{i}\left(x\right)|\leq \beta_{i}\left|x|,\;\;\;\;\right|H_{i}\left(x\right)|\leq \gamma_{i}\left|x\right|,\;\;x,y\in R.$$

### 5.3. Stability of the Model by Lyapunov Functions and Razumikhin Method

Note that the singularity of the solutions of the differential equations with GRLFD and RLFD at the initial time requires this point to be excluded and for some stability properties to be obtained on an interval without the initial time. It is totally different than the case of Caputo type fractional derivative or derivative with an integer order. Some authors apply RLFD but do not exclude the initial time and fact that for order α∈(0,1). The expressions $t^{-\alpha }$ and $t^{\alpha -1}$ are not bounded for points enough close to the initial time 0 (see, for example, [6,25,26]). The main concepts of stability for differential equations with RLFD are discussed and studied in [27].

In connection with the applied GRLFD, we need to define the exponential stability of the equilibrium on an interval excluding the initial time 0.

**Definition 4.**  *The constant equilibrium*$V^{*}$of (40) is called exponentially stable in time if there exist a numberC>0*, a point* T>0*, and an increasing function*$\Xi \in C(R_{+},R_{+})$*such that any solution*y(t))*of (40), (30) satisfies*

$$∥y\left(t\right)-V^{*}∥\leq \Xi \left({∥\phi ∥}_{0}\right)e^{-C|\lambda -1|t},\;t\geq T.$$

Note that if a function is RL integrable (or generalized proportional RL integrable), it is not necessarily to be squared RL integrable (or squared generalized proportional RL integrable).

**Example 1.**  *Let ρ=0.5,q=0.3, and $\nu \left(t\right)=t^{0.8}$. Then, the integrals $\int_{0}^{1}e^{-\left(1-s\right)}{\left(1-s\right)}^{-0.3}s^{-0.8}\;ds$ and $\int_{0}^{1}{\left(1-s\right)}^{-0.3}s^{-0.8}\;ds$ exist but the corresponding integrals of the squared function $u^{2}\left(t\right)=t^{-1.6}$, i.e., $\int_{0}^{1}e^{-\left(1-s\right)}{\left(1-s\right)}^{-0.3}s^{-1.6}\;ds$ and $\int_{0}^{1}{\left(1-s\right)}^{-0.3}s^{-1.6}\;ds$, do not exist.*

It is necessary, in the application of the squared Lyapunov function as well the product $u^{T}\left(t\right)u\left(t\right)$ for the solution u(t) of the model (40), to assume that any solution is squared RL integrable (or squared generalized proportional RL integrable) on the whole time interval of consideration. This is a huge restriction regarding the solutions of the model (for example, see Theorems 3.1 and 3.2 [24] where Lyapunov functional $V_{1}$ is used without assuming squared integrability of the solution).

Now we will apply quadratic Lyapunov functions to study the stability properties of the model (40), (30).

**Theorem 2.**  *Let the assumptions A1–A4 be satisfied and*

1. The fractional neural model (40) has an equilibrium $V^{*}=\left(C_{1},C_{2},\ldots ,C_{n}\right)$.
2. *Any solution $u\in C_{\alpha ,\lambda }\left(\left[0,\infty \right),R^{n}\right)$ is squared fractional integrable, i.e., $u^{T}u\in C_{\alpha ,\lambda }\left(\left[0,\infty \right),R^{n}\right)$.*
3. *For all t≥0, the inequalities*
$$\begin{array}{cc} & \sum_{k=1}^{n}\{\left(\max_{i=1,2,\ldots ,n}|a_{i,k}\left(t\right)|+\max_{i=1,2,\ldots ,n}\left|a_{k,i}\left(t\right)\right|\right)\alpha +\left(\max_{i=1,2,\ldots ,n}|b_{i,k}\left(t\right)|+\max_{i=1,2,\ldots ,n}\left|b_{k,i}\left(t\right)\right|\right)\beta \\ & +\left(\max_{i=1,2,\ldots ,n}|c_{i,k}\left(t\right)|+\max_{i=1,2,\ldots ,n}\left|c_{k,i}\left(t\right)\right|\right)\tau \gamma \}<2\min\left\{\mu_{i},\;i=1,2,\ldots ,n\right\} \end{array}$$
  *hold, where $\alpha =\max_{i=1,2,\ldots ,n}\alpha_{i}$, $\beta =\max_{i=1,2,\ldots ,n}\beta_{i}$, and $\gamma =\max_{i=1,2,\ldots ,n}\gamma_{i}.$*

Then, the equilibrium $V^{*}$ is exponentially stable in time, i.e., there exists a point $T_{\alpha }>0$ such that any solution $u\in C_{\alpha ,\lambda }\left(\left[0,\infty \right),R^{n}\right)$ of (40), (30) satisfies the inequality

$$||u\left(t\right)-V^{*}\left|\right|<\frac{\sum_{i=1}^{n}\max_{t\in [\tau ,0]}\left|\phi_{i}\left(t\right)\right|\;\rho^{1-\alpha }}{\Gamma (q)}e^{-|\lambda -1|t}\;for\;t>T_{\alpha }.$$

**Proof.**  Consider the Lyapunov function $V\left(x\right)=0.5x^{T}x=0.5\sum_{i=1}^{n}x_{i}^{2},\;x\in R^{n},\;x=\left(x_{1},x_{2},\ldots ,x_{n}\right)$.

Let $u\left(t\right)\in C_{\alpha ,\lambda }\left(\left[0,\infty \right),R^{n}\right)$ be a solution of (40), (30). Consider the system (10) with initial conditions (30). We will study the stability of its zero solution. Let $\nu_{i}\left(t\right)=u_{i}\left(t\right)-C_{i},\;i=1,2,\ldots ,n.$ Then, $\nu \in C_{\alpha ,\lambda }\left(\left[0,\infty \right),R^{n}\right)$ is a solution of (10), (30) and ${}_{0}^{RL}D_{t}^{\alpha ,\lambda }V\left(\nu \left(t\right)\right)=0.5\sum_{i=1}^{n}\;{}_{0}^{RL}D_{t}^{\alpha ,\lambda }\nu_{i}^{2}\left(t\right),\;t>0.$

Let point t>0 be such that $\sum_{i=1}^{n}\nu_{i}^{2}\left(t+\sigma \right)<\sum_{i=1}^{n}\nu_{i}^{2}\left(t\right)$ for σ∈[−min{τ,t},0], i=1,2,…,n.

Apply Corollary 1, assumptions A1–A3, inequality $ab\leq 0.5a^{2}+0.5b^{2}$, and we obtain

$$\begin{array}{cc} & {}_{0}^{RL}D_{t}^{\alpha ,\lambda }V\left(\nu \left(t\right)\right)\leq -\sum_{i=1}^{n}\mu_{i}\nu_{i}^{2}\left(t\right)+0.5\sum_{i=1}^{n}\nu_{i}^{2}\left(t\right)\sum_{k=1}^{n}|a_{i,k}\left(t\right)|\alpha_{k}+0.5\sum_{i=1}^{n}\sum_{k=1}^{n}\left|a_{i,k}\left(t\right)\right|\alpha_{k}\nu_{k}^{2}\left(t\right)\\ & +0.5\sum_{i=1}^{n}\nu_{i}^{2}\left(t\right)\sum_{k=1}^{n}|b_{i,k}\left(t\right)|\beta_{k}+0.5\sum_{i=1}^{n}\sum_{k=1}^{n}\left|b_{i,k}\left(t\right)\right|\beta_{k}\nu_{k}^{2}\left(t-\xi \left(t\right)\right)\\ & +0.5\sum_{i=1}^{n}\sum_{k=1}^{n}|c_{i,k}\left(t\right)|\nu_{i}^{2}\left(t\right)\Theta \left(t\right)\gamma_{k}+0.5\sum_{i=1}^{n}\sum_{k=1}^{n}\left|c_{i,k}\left(t\right)\right|\int_{t-\Theta (t)}^{t}\gamma_{k}\nu_{k}^{2}\left(s\right)ds\\ & \leq -\sum_{i=1}^{n}\mu_{i}\nu_{i}^{2}\left(t\right)+0.5\sum_{i=1}^{n}\nu_{i}^{2}\left(t\right)\sum_{k=1}^{n}\max_{i=1,2,\ldots ,n}|a_{i,k}\left(t\right)|\alpha +0.5\sum_{i=1}^{n}\nu_{i}^{2}\left(t\right)\sum_{k=1}^{n}\max_{i=1,2,\ldots ,n}\left|a_{k,i}\left(t\right)\right|\alpha \\ & +0.5\sum_{i=1}^{n}\nu_{i}^{2}\left(t\right)\sum_{k=1}^{n}\max_{i=1,2,\ldots ,n}|b_{i,k}\left(t\right)|\beta +0.5\sum_{i=1}^{n}\nu_{i}^{2}\left(t-\xi \left(t\right)\right)\sum_{k=1}^{n}\max_{i=1,2,\ldots ,n}\left|b_{k,i}\left(t\right)\right|\beta \\ & +0.5\sum_{i=1}^{n}\nu_{i}^{2}\left(t\right)\sum_{k=1}^{n}\max_{i=1,2,\ldots ,n}|c_{i,k}\left(t\right)|\tau \gamma +0.5\int_{t-\Theta (t)}^{t}\sum_{i=1}^{n}\nu_{i}^{2}\left(s\right)ds\sum_{k=1}^{n}\max_{i=1,2,\ldots ,n}\left|c_{k,i}\left(t\right)\right|\gamma \\ & \leq 0.5\sum_{i=1}^{n}(-2\mu_{i}+\sum_{k=1}^{n}(\max_{i=1,2,\ldots ,n}|a_{i,k}\left(t\right)|\alpha +\max_{i=1,2,\ldots ,n}\left|a_{k,i}\left(t\right)\right|\alpha \\ & +\max_{i=1,2,\ldots ,n}|b_{i,k}\left(t\right)|\beta +\max_{i=1,2,\ldots ,n}|b_{k,i}\left(t\right)|\beta +\max_{i=1,2,\ldots ,n}|c_{i,k}\left(t\right)|\tau \gamma +\tau \max_{i=1,2,\ldots ,n}\left|c_{k,i}\left(t\right)\right|\gamma ))\nu_{i}^{2}\left(t\right)\\ & <0. \end{array}$$

From Corollary 3, the claim of Theorem 2 follows. □

**Corollary 4.**  *Let the conditions of Theorem 2 be satisfied. Then, any solution $u\in C_{\alpha ,\lambda }\left(\left[0,\infty \right),R^{n}\right)$ of (40), (30) satisfies $\lim_{t\to \infty }u_{i}\left(t\right)=C_{i},\;i=1,2,\ldots ,n,$ i.e., any solution of the model (40) approaches the equilibrium at infinity.*

## 6. Applications

**Example 2.**  *Consider the following neural networks with three neurons with the GRLFD:*

(43) $$\begin{array}{cc} & {}_{0}^{RL}D_{t}^{0.3,0.5}u_{i}\left(t\right)=-A_{i}\left(t\right)u_{i}\left(t\right)+\sum_{k=1}^{3}a_{i,k}\left(t\right)f_{k}\left(u_{k}\left(t\right)\right)\\ & +\sum_{k=1}^{3}b_{i,k}\left(t\right)g_{k}\left(u_{k}\left(t-2\right)\right)+\sum_{k=1}^{3}c_{i,k}\left(t\right)\int_{t-e^{-t}}^{t}h_{k}\left(u_{k}\left(s\right)\right)ds+I_{i}\left(t\right),\;\;t>0,\;\;i=1,2,3, \end{array}$$

*with λ=0.5,α=0.3, $\xi \left(t\right)=2,\;\Theta \left(t\right)=e^{-t}$, τ=2, coefficients $A_{1}\left(t\right)=2,\;A_{2}\left(t\right)=0.5e^{t},\;\;A_{3}\left(t\right)=1+0.5e^{t}$, and, therefore, $\mu_{1}=2,\;\mu_{2}=0.5,\;\mu_{3}=1.5$.*

*The activation functions $f_{1}\left(x\right)=g_{1}\left(x\right)=h_{1}\left(x\right)=\frac{x}{1+e^{-x}}$ are the Swish functions with constants $\alpha_{1}=\beta_{1}=\gamma_{1}=1.1$, $f_{2}\left(x\right)=g_{2}\left(x\right)=h_{2}\left(x\right)=\frac{e^{x}-e^{-x}}{e^{x}+e^{-x}}$ are the tanh functions with constants $\alpha_{2}=\beta_{2}=\gamma_{2}=1$, and $f_{3}\left(x\right)=g_{3}\left(x\right)=h_{3}\left(x\right)=0.5\left(|x+1|-|x-1|\right)$ with $\alpha_{3}=\beta_{3}=\gamma_{3}=1$, the external inputs are given by*

$$I_{1}\left(t\right)=2+\frac{0.5^{0.5}}{\Gamma (0.7)}\left(e^{0.5t}t^{-0.3}+0.5^{0.3}\gamma \left(0.5,0.5t\right),\right),\;I_{2}\left(t\right)=0.1e^{-t}\frac{e}{1+e},\;\;I_{3}\left(t\right)=0.001\frac{e}{1+e}$$

*and the matrices of strengths of interconnections*

$$\left\{a_{i,k}\left(t\right)\right\}=\left[\begin{array}{ccc} 0.01 & 0 & 0.02\\ 0.01e^{-t} & 0.01 & 0\\ 0 & \frac{0.01t}{1+t} & 0 \end{array}\right],\;\;\;\;\left\{b_{i,k}\left(t\right)\right\}=\left[\begin{array}{ccc} -0.01 & 0.1 & 0.1\\ -0.1e^{-t} & 0 & 0.1e^{-2t}\\ 0 & 0.001\sin(t) & 0 \end{array}\right],$$

$$\left\{c_{i,k}\left(t\right)\right\}=\left[\begin{array}{ccc} 0 & 0.01 & -0.01e^{-t}\\ -0.01 & \frac{0.0022t}{1+t} & 0\\ -0.001 & 0 & 0.005e^{-t}\; \end{array}\right]$$

*with $\sum_{k=1}^{3}\max_{i=1,2,3}\left|b_{k,i}\left(t\right)\right|=0.1+0.1e^{-t}+0.05\sin\left(t\right)\leq 0.25$ and $\sum_{k=1}^{3}\max_{i=1,2,3}\left|c_{k,i}\left(t\right)\right|=0.01+0.01+0.005e^{-t}\leq 0.025$.*

*Then, the inequality*

$$\begin{array}{cc} & \sum_{k=1}^{3}\{\left(\max_{i=1,2,3}|a_{i,k}\left(t\right)|+\max_{i=1,2,3}\left|a_{k,i}\left(t\right)\right|\right)1.1+\left(\max_{i=1,2,3}|b_{i,k}\left(t\right)|+\max_{i=1,2,3}\left|b_{k,i}\left(t\right)\right|\right)1.1\\ & +\left(\max_{i=1,2,3}|c_{i,k}\left(t\right)|+\max_{i=1,2,3}\left|c_{k,i}\left(t\right)\right|\right)2.2\}\\ & \leq \left(0.04+0.04\right)1.1+\left(0.25+0.3\right)1.1+\left(0.025+0.025\right)2.2=0.803<2\min\left\{\mu_{1},\mu_{2},\mu_{3}\right\}=1 \end{array}$$

*is satisfied, i.e., condition 3 of Theorem 2 is satisfied.*

The model (43) has an equilibrium $V^{*}=\left(1,0,0\right)$ because $f_{1}\left(1\right)=g_{1}\left(1\right)=h_{1}\left(1\right)=\frac{e}{1+e}$, $f_{2}\left(0\right)=g_{2}\left(0\right)=h_{2}\left(0\right)=0$, $f_{3}\left(0\right)=g_{3}\left(0\right)=h_{3}\left(0\right)=0$ and the equalities

(44) $$\begin{array}{cc} & \frac{0.5^{0.5}}{\Gamma (0.7)}\left(e^{0.5t}t^{-0.3}+0.5^{0.3}\gamma \left(0.5,0.5t\right)\right)=-A_{1}\left(t\right)+\left(a_{1,1}\left(t\right)+b_{1,1}\left(t\right)+c_{1,1}\left(t\right)e^{-t}\right)\frac{e}{1+e}+I_{1}\left(t\right),\\ & 0=\left(a_{2,1}\left(t\right)+b_{2,1}\left(t\right)+c_{2,1}\left(t\right)e^{-t}\right)\frac{e}{1+e}+I_{2}\left(t\right)\\ & 0=\left(a_{3,1}\left(t\right)+b_{3,1}\left(t\right)+c_{3,1}\left(t\right)e^{-t}\right)\frac{e}{1+e}+I_{3}\left(t\right)\;\;\;\;\;\;for\;\;t\geq 0 \end{array}$$

*hold.*

*According to Theorem 2, the equilibrium of (43) is exponentially stable in time, i.e., every solution $\left(u_{1}\left(\cdot \right),u_{2}\left(\cdot \right),u_{3}\left(\cdot \right)\right)$ of (43) is satisfying the inequality*

$$\sqrt{u_{1}{\left(t\right)-1)}^{2}+u_{2}^{2}\left(t\right)+u_{3}^{2}\left(t\right)}\leq \sum_{i=1}^{3}\max_{t\in [-2,0]}\left|\phi_{i}\left(t\right)\right|\frac{0.5^{0.7}e^{-t}}{\Gamma (0.3)},\;\;t>T=1,$$

*where $T:t^{-0.7}=1$.*

## 7. Conclusions

The main aim of the paper is to study Hopfield neural networks with both the variable delay and distributed delay. An important aspect of our study is that we consider the general case of variables in time coefficients and external inputs. The dynamic of the units are modeled by the appropriately defined generalized fractional derivatives of Riemann–Liouville type, satisfying the first and the second fundamental theorem in fractional calculus. This derivative is applied to adequately model the behavior with anomalies at the initial time point. An exponential type of stability is defined and this stability excludes the initial time because of the singularity of the solutions at the initial time. Quadratic Lyapunov functions and Razumikhin method are applied. Theoretical results are illustrated with an example. The main inequality for the general convex Lyapunov functions could be applied to theoretical study of stability behavior of fractional differential equations with the applied generalized fractional derivative as well to study the stability equilibrium of many models with this type of fractional derivative.

## Data Availability

Not applicable.
